# Head-down tilt bed rest with or without artificial gravity is not associated with motor unit remodeling

**DOI:** 10.1007/s00421-020-04458-7

**Published:** 2020-08-14

**Authors:** Julia Attias, Andrea Grassi, Alessandra Bosutti, Bergita Ganse, Hans Degens, Michael Drey

**Affiliations:** 1 Research Centre for Musculoskeletal Science & Sports Medicine, Manchester Metropolitan University, Manchester, UK; 2 Schulthess Clinic, Zurich, Switzerland; 3 Department of Life Sciences, University of Trieste, Trieste, Italy; 4 Lithuanian Sports University, Kaunas, Lithuania; 5 Department of Medicine IV, University Hospital, LMU Munich, Munich, Germany

**Keywords:** Motor unit, Bed rest, Neuromuscular, MUNIX, Microgravity, Spaceflight, Muscle strength

## Abstract

**Purpose:**

The objective of this study was to assess whether artificial gravity attenuates any long-duration head-down 6^0^ bed rest (HDBR)-induced alterations in motor unit (MU) properties.

**Methods:**

Twenty-four healthy participants (16 men; 8 women; 26–54 years) underwent 60-day HDBR with (*n* = 16) or without (*n* = 8) 30 min artificial gravity daily induced by whole-body centrifugation. Compound muscle action potential (CMAP), MU number (MUNIX) and MU size (MUSIX) were estimated using the method of Motor Unit Number Index in the Abductor digiti minimi and tibialis anterior muscles 5 days before (BDC-5), and during day 4 (HDT4) and 59 (HDT59) of HDBR.

**Results:**

The CMAP, MUNIX, and MUSIX at baseline did not change significantly in either muscle, irrespective of the intervention (*p* > 0.05). Across groups, there were no significant differences in any variable during HDBR, compared to BDC-5.

**Conclusion:**

Sixty days of HDBR with or without artificial gravity does not induce alterations in motor unit number and size in the ADM or TA muscles in healthy individuals.

## Introduction

Exposure to microgravity induces a myriad of physiological alterations (Mulavara et al. 2018). Head-down bed rest (HDBR) is an accepted earth-based model of the microgravity experienced during spaceflight (Pave-Le Traon et al. 2007). Both spaceflight and HDBR are accompanied with a significant loss in muscle strength, which is a major concern for astronauts during long-term space missions (Rittweger et al. 2018). Maintenance of muscle strength during spaceflight is crucial, not only because crew are required to perform physical work during extravehicular activities, but also to ensure a prompt return to one’s functional capacity and performance of daily, steady motor tasks following flight and bed rest (Clark et al. 2007; Vinstrup et al. 2017).

In HDBR, loss of muscle strength is proportionally larger than the loss of muscle mass (Belavý et al. 2009; Alkner and Tesch 2004), and occurs particularly during the early phases of bed rest (Campbell et al. 2019). For instance, Kawakami et al. (2001) reported that the 10.9% reduction in knee extension force after 20 days of bed rest was greater than the 7.8% decrease in cross-sectional area, though proportional to decreases in voluntary activation. Furthermore, it has been shown that the neuromuscular activation during walking is altered after long-duration spaceflight (Layne et al. 1998) and such changes may be a consequence of changes in the neuromuscular junction (NMJ). In line with this, a number of morphological changes in the NMJs in rat leg muscles have been observed during spaceflight lasting ≥ 16 days, including nerve terminal sprouting (D’Amelio and Daunton 1992) and NMJ expansion (Baranski and Marciniak 1979) possibly resulting from denervation (Riley et al. 1990). These adaptations may cause MU remodeling, such as an increase in size. Interestingly, NMJ degeneration has also been reported in ageing mice, with a concomitant decline in motor unit (MU) number, where the loss of MUs could be prevented by exercise, suggesting a functional rather than anatomical loss of MUs (Giorgetti et al. 2019). Such a situation may also occur in humans exposed to microgravity, seeing as fundamental MU organization, i.e., presence of a motor neuron and muscle fibres innervated by it, is comparable to that of most mammals (Purves et al. 2001).

Indeed, it was argued by Kawakami et al. (2001) that the force decrements during bed rest resulted from decreased motor neuron excitability and impaired MU activation. Maximal MU firing rates have been significantly reduced in the first dorsal interosseous muscle after 6 weeks of immobilization in healthy individuals (Seki et al. 2001). Moreover, 1 day of leg casting was enough to induce reductions in MU number in the Vastii group (Fuglsang-Frederiksen and Scheel 1978), as suggested by the reduced integrated electromyography (EMG) during a maximal voluntary contraction (MVC). It has been shown that the muscle fibre conduction velocity (MFCV) is positively related to the twitch torque and negatively related to the contraction and relaxation times of an MU (Andreassen, and Arendt-Nielsen 1987). A decrease in MFCV in the tibialis anterior muscle (TA; ~ 5–10%) and vastus medialis (7–13%) muscles (Cescon and Gazzoni 2010) has been observed during a 14d-day bed rest, suggesting that there may be some MU remodeling occurring. Nonetheless, any claims related to MU parameters during these studies can only be speculative, given the absence of a method of direct assessment. Moreover, equivalent information of healthy individuals undergoing long periods of whole-body disuse is unavailable at present.

The “motor unit number index” (MUNIX) method (Nandedkar et al. 2003) is an easy, non-invasive, and time-efficient method that has been validated against other MU number estimation (MUNE)-based models. Previous testing with this method has shown a good inter and intra-rater reproducibility in determining MU number and size of hand and leg muscles and reliable detection of progressive changes in these parameters during diseases, such as Amyotrophic Lateral Sclerosis (Drey et al. 2013; Fathi et al. 2016; Neuwirth et al. 2011, 2016). Thus, the MUNIX method has utility to explore the time-course changes of MU adaptations.

The most obvious solution to tackle physiological adaptations resulting from microgravity is to recreate the element that is lost. As such, artificial gravity (AG) has been proposed in the form of human centrifugation for ~ 20 years (Kreitenberg et al. 1998), which has been employed as a countermeasure during short-duration (5 days) HDBR (Rittweger et al. 2015). It was shown that short bouts of intermittent AG with the same duration of AG exposure in one single bout were better tolerated (Vernikos et al. 1996) and improved isometric strength of the knee extensors and flexors (Rittweger et al. 2015). Therefore, AG may be a promising countermeasure to maintain the neuromuscular system during long-term microgravity and HDBR-induced disuse.

The objectives of this study were (a) to assess the effect of long-duration HDBR on MU number and size in both upper and lower body muscles and (b) to determine the efficacy of AG to attenuate any bed rest-induced alterations in MU number or size. We hypothesized that (1) MU number decreases and size increases with HDBR and to a greater extent in the muscles of the lower body compared to those in the upper body, due to the load-bearing function of the latter, and (2) that (a) AG prevents any such deleterious effects on MU number and size, where (b) intermittent AG is superior to continuous AG.

## Methods

### Experimental design

Twenty-four healthy individuals (16 men and 8 women; 33.3 ± 9.2 years; 175 ± 9 cm; 74.2 ± 10 kg) consented to and were confined to 60-day head-down bed rest (HDBR) as part of a joint study of the European Space Agency (ESA) and the National Aeronautics and Space Administration (NASA), called the Artificial Gravity Bed Rest—European Space Agency (AGBRESA) study. The study took place at the German Aerospace Centre in Cologne, Germany, between March and December of 2019.

The study was organized in two campaigns, each of which consisted of 15 days for baseline data collection (BDC-15 to BDC-1), 60 days of HDBR (HDT1-HDT59), and 14 days of recovery (R + 0 to R + 13). The study was approved by the ethics committee (2018143) of the North Rhine Medical Association (Ärztekammer Nordrhein) in Düsseldorf, Germany, and was registered in the German Clinical Trials Register (DRKS-ID: DRKS00015677).

Sixteen of the participants received 30 min of AG daily via human centrifugation and the other eight served as a control group (6 men, 2 women). The AG group was further divided into two subgroups: those receiving AG in one continuous 30-min bout (AG1; *n* = 8; 5 men and 3 women) or in 6 × 5-min bouts with 3-min rest in between bouts (AG2; *n* = 8; 5 men and 3 women). For the first campaign, participants were pseudo-randomly assigned to groups, whereas for campaign 2, assignment was based on demographic balancing particularly with regards to sex, due to the drop out of three women and subsequent replacement during campaign 2.

Each participant received a complete description of the experimental methods and passed the medical and psychological screening criteria. Medical tests for selection were similar to those used in a previous ESA HDBR study (Rittweger et al. 2015). In addition, the participants passed a centrifuge tolerance test (AG2 protocol) to confirm their eligibility. All participants were made aware of their right to withdraw from the experiment at any time.

### Head-down bed rest (HDBR)

Six-degree HDBR was performed in conformity with the International Guidelines for Standardization of bed rest studies in the spaceflight context (https://www.nasa.gov/sites/default/files/atoms/files/bed_rest_studies_complete.pdf). Participants followed a day–night cycle of 06:30 a.m. wake-up, and 22:00 p.m. lights-out. During the HDT period, strict bed rest was followed, and all activities were conducted in the HDT position (hygiene, toilet, reading, etc.). Participants were allowed to change position during HDT, ensuring that one shoulder was in contact with the bed at all times, but not to get up, sit, or stand. During this period, participants were monitored by video and staff surveillance to ensure compliance with the protocol. During the ambulatory and rehabilitation periods, the participants were not authorized to leave the research facility. During their free time, they were allowed leisure activities such as reading, playing games, or computer activities. Daytime sleeping or napping was not permitted. The temperature of the residential area within the facility was controlled across both campaigns at around 22.5 °C (Campaign 1: 22.7 ± 1.6 °C; Campaign 2: 22.5 ± 1.6 °C).

### Application of short-arm centrifugation

Transfer to the centrifuge was accomplished with a specific gurney, so that the participants remained at − 6° during transport and were then asked to roll over to the centrifuge nacelle without using their leg muscles. During centrifugation, participants were exposed to 1 g at their estimated centre of mass and were instructed to stay calm, to not move the head, and to keep their leg muscles relaxed. A centrifuge run was as follows: acceleration at 5° s^−2^ for 32–33 s until the target rotation speed was achieved. Rotation at constant velocity then lasted either 30 min (AG1) or 5 min, with a 3-min rest, repeated six times (AG2). After each run, deceleration was at 5° s^−2^ until a complete stop. Continuous medical monitoring to ensure participant safety was implemented during all centrifuge runs. The time-of-day for centrifugation was randomly assigned on a day-to-day basis.

### Estimation of motor unit number and size

**Fig. 1:**
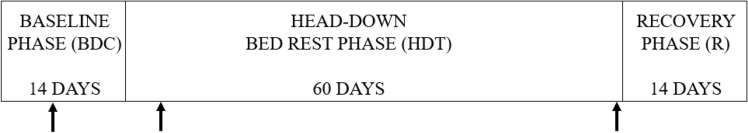
Timeline of measurements. CMAP, MUNIX, and MUSIX measurements were taken 5 days prior to the beginning of the bed rest phase (BDC-5), day 4 into head-down bed rest (HDT4), and 59 days into head-down bed rest (HDT59). No measurements were taken during the recovery phase

Measurements of both MUNIX and MUSIX were made from the *m. abductor digiti minimi* (ADM) and *m. tibialis anterior* (TA) on day 5 before HDBR (BDC-5) and on days 4 (HDT4) and 59 (HDT59) of HDBR (Fig. 1). No measurements were taken during the recovery period. First, the compound muscle action potential (CMAP) was recorded (Viking on Nicolet EDX electromyograph, V22, Natus Neurology, Middleton, Wisconsin, USA), followed by the SIP recordings, at a sampling rate of 50 kHz. A 5–1000-Hz band-pass filter setting was employed to offer a stable baseline and eliminate high-frequency noise. For both muscles, the position of the active recording electrode (15 mm × 20 mm; CareFusion, Middleton, Wisconsin, USA) was adjusted until the highest CMAP amplitude was obtained during BDC-5. This position was marked and noted to minimize errors related to differences in electrode placement between subsequent testing sessions. For the ADM, the active electrode was placed over the motor point of the right hypothenar muscle and the reference electrode at the distal phalanx of the little finger. We used cellophane sheets and a permanent marker to mark the position and other skin landmarks, i.e., palmar creases that could be placed over the hand. The position of the active TA electrode was marked taking the cross-point of the vertical distance from the lateral malleolus and the horizontal distance from the tibial crest, and the reference electrode was placed on the patella. Finally, the ground electrode was placed ~ 2 cm under the stimulation point of the ulnar nerve and ~ 5 cm above the ankle malleoli, for the ADM and TA, respectively. The fingers and toes were strapped to inhibit any dynamic movement. Both the ulnar and peroneal nerves were stimulated with rectangular pulses of 0.2 ms, starting at 10 mA and increasing in 5 mA increments until maximal CMAP was achieved. Following CMAP acquisition, the participant was instructed to produce a number of voluntary isometric contractions (VICs) of the respective muscle. Upon instruction, the participant first produced a contraction with maximal effort, termed 10/10, followed by the minimum contraction necessary to create the desired action, i.e., finger abduction or dorsiflexion, termed 1/10. Participants were then asked to cover the range of forces in between, i.e., 2/10, 3/10, etc., ensuring that each number was covered at least twice, with short rests in between. The force was not measured, though, for each contraction, the surface interference pattern (SIP) of the EMG trace was recorded, with each epoch lasting 300 ms.

### Data analysis

The CMAP and SIP signals were used to calculate the signals’ area and power (Nandedkar et al. 2004; Viking on Nicolet EDX electromyograph, Natus Neurology). The ‘‘ideal case motor unit count’’ (ICMUC) is computed using Eq. (5) in “Appendix” (Drey et al. 2013). The relationship between ICMUC and SIP area (${\text{ICMUC}} = A \cdot ({\text{Area}}({\text{SIP}}))^{\alpha }$) is modelled by a power function. The values of $A$ and $\alpha$ are derived from a regression analysis fitting the power function. The regression curve characterizes the tested muscle. Finally, MUNIX is calculated by:

$$ {\text{MUNIX}} = A \cdot (20\,{\text{mVms}})^{\alpha } . $$

An SIP area value of 20 mVms is chosen, based on (1) the observation that very slight activity, produced by a few motor units, has an SIP area of around 20 mVms (Nandedkar et al. 2010), and (2) the fact that the assumptions of the model are adequately satisfied for an SIP area of 20 mVms.

MUSIX is obtained by dividing the CMAP amplitude by MUNIX: ${\text{MUSIX}} = \frac{{{\text{Amplitude}}({\text{CMAP}})}}{\text{MUNIX}}.$

It should be stressed that MUNIX and MUSIX are indices, and not absolute values, for the number and size of MUs. Any within-session variation in CMAP > 10% was excluded from analysis. For all variables, data from BDC-5 are represented as mean ± SD. To compare differences between groups, data at BDC-5 were normalized per participant to their group mean, and data from HDT4 and HDT59 were calculated as a percent change from BDC-5.

### Statistical analysis

Normal distribution of data was confirmed by the Kolmogorov–Smirnov test. A one-way ANOVA was used to assess participant characteristics at baseline for differences in age, sex (chi-squared test), height, and weight between groups. Repeated-measures ANOVA was used to determine the change in CMAP, MUNIX, and MUSIX at HDT4 and HDT59 compared to BDC-5, with TIME as a within-participant factor and GROUP as a between-participant factor. TIME * GROUP interactions were also determined. Post hoc tests used a Bonferroni correction to account for testing of multiple pairs. Significance was defined as *p* < 0.05.

## Results

### Baseline characteristics

There were no significant differences in the age, sex distribution, height, and body mass of the participants between groups at BDC-5 (Table 1). Additionally, the group means for CMAP, MUNIX, and MUSIX did not differ significantly for the ADM or the TA (Table 1).

**Table 1:** Participant characteristics per group at BDC-5

Variable	Control	AG1	AG2	Group difference *p* values^α^
*n*	8	8	8	
Age (years)	32.3 ± 7.4	33.5 ± 10.9	34.0 ± 10.1	*p* = 0.932
Sex	6 men; 2 women	5 men; 3 women	5 men; 3 women	*p* = 0.829*
Height (cm)	177 ± 7	172 ± 8	174 ± 11	*p* = 0.598
Body mass (kg)	79.4 ± 12.7	71.8 ± 10.2	71.4 ± 4.5	*p* = 0.203
Body mass index (kg/m^2^)	25.2 ± 2.6	24.0 ± 1.7	23.6 ± 1.6	*p* = 0.273
ADM				
CMAP (mV)	13.0 ± 2.2	13.6 ± 3.1	12.8 ± 1.9	*p* = 0.817
MUNIX	215 ± 46	196 ± 40	208 ± 45	*p* = 0.700
MUSIX (µV)	62 ± 11	71 ± 14	64 ± 13	*p* = 0.410
TA				
CMAP (mV)	10.0 ± 2.1	10.4 ± 2.5	9.0 ± 1.3	*p* = 0.390
MUNIX	234 ± 48	237 ± 66	219 ± 37	*p* = 0.760
(MUSIX; µV)	43 ± 5	45 ± 5	43 ± 9	*p* = 0.769

*ADM* abductor digiti minimi, *TA* tibialis anterior, *CMAP* compound muscle action potential, *MUNIX* motor unit number index, *MUSIX* motor unit size index; ^α^one-way ANOVA; *Chi-square test. Data are mean (± SD)

The effect sizes and significance values for TIME and GROUP are reported in Table 2. The results for each parameter will be described in detail below.

**Table 2:** The effect size and *p* values for the main and interaction effects for the ADM and TA

Variable	Time Effect size; *p* value	Group Effect size; *p* value	Time * group Effect size; *p* value
ADM			
(CMAP)	0.006; *p* = 0.889	0.029; *p* = 0.737	0.020; *p* = 0.929
(MUNIX)	0.075; *p* = 0.196	0.073; *p* = 0.450	0.035; *p* = 0.818
(MUSIX)	0.094; *p* = 0.125	0.034; *p* = 0.696	0.027; *p* = 0.884
TA			
(CMAP)	0.039; *p* = 0.430	0.009; *p* = 0.907	0.003; *p* = 0.998
(MUNIX)	0.006; *p*-0.889	0.098; *p* = 0.340	0.030; *p* = 0.861
(MUSIX)	0.026; *p* = 0.570	0.090; *p* = 0.372	0.026; *p* = 0.886

*ADM* abductor digiti minimi, *TA* tibialis anterior, *CMAP* compound muscle action potential, *MUNIX* motor unit number index, *MUSIX* motor unit size index; *n* = 24

### CMAP

**Fig. 2:**
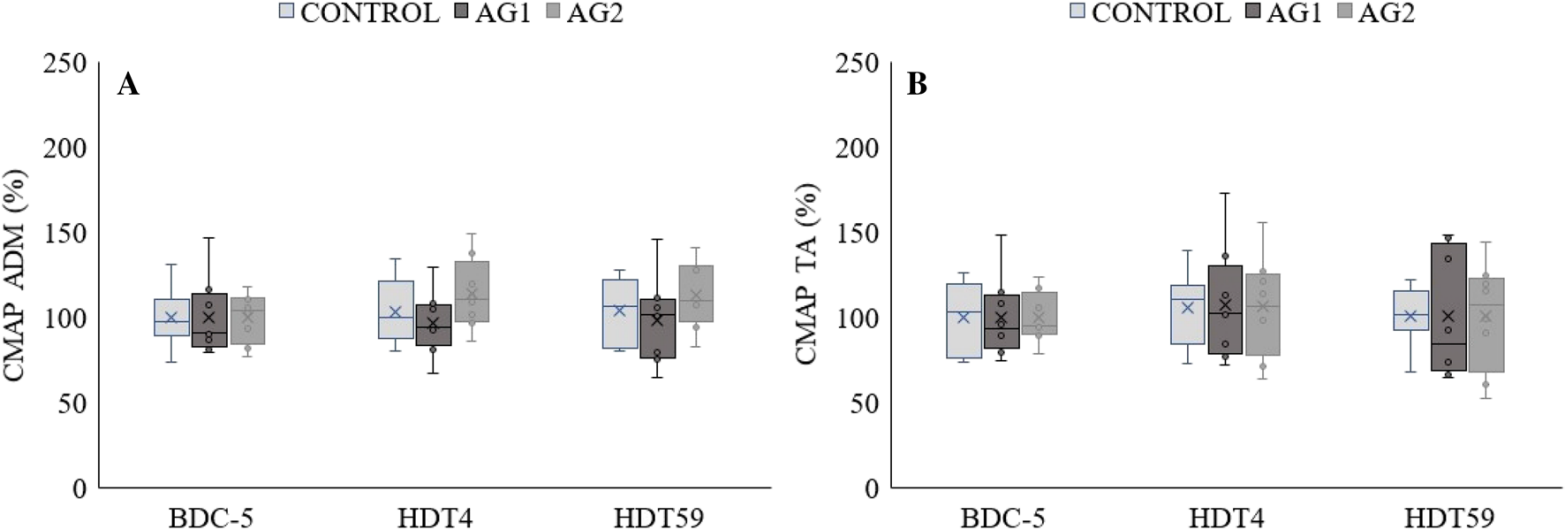
Compound muscle action potential (CMAP) of the **a** *m. adductor digiti minimi* (ADM) and **b** *m. tibialis anterior* (TA) before (BDC-5), and at day 4 (HDT4) and 59 (HDT59) of bed rest in CONTROL, AG1 (bed rest + 30 min daily centrifugation) and AG2 (bed rest + 6 × 5 min daily centrifugation). Absolute values from BDC-5 are divided by the group mean to represent 100%. The box plots represent the minimum, first quartile, median, third quartile, and maximum; the horizontal line reflect the mean of the respective group. Dots represent outliers. *n* = 8 per group

In both the ADM (Fig. 2a) and TA (Fig. 2b), there were no significant changes in CMAP at HDT4 and HDT59 compared to BDC-5 in any of the groups.

### MUNIX

**Fig. 3:**
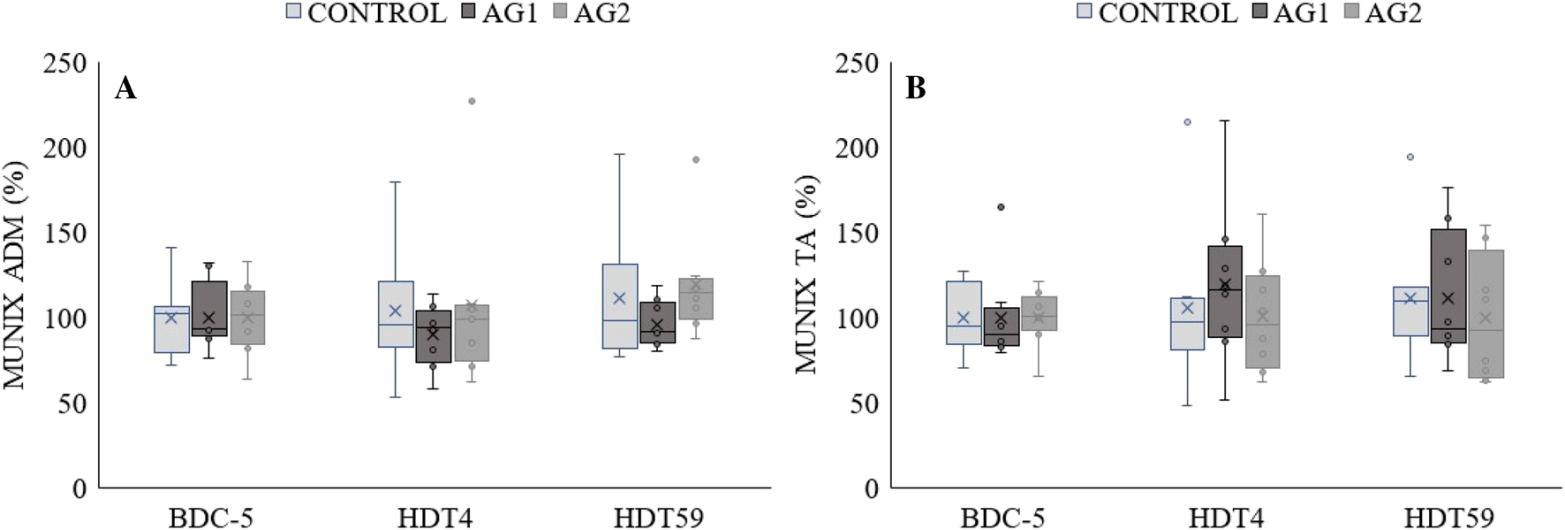
Motor unit number (MUNIX) of the **a** *m. adductor digiti minimi* (ADM) and **b** *m. tibialis anterior* (TA) before (BDC-5), and at day 4 (HDT4) and 59 (HDT59) of bed rest in CONTROL, AG1 (bed rest + 30 min daily centrifugation) and AG2 (bed rest + 6 × 5 min daily centrifugation). Absolute values from BDC-5 are divided by the group mean to represent 100%. The box plots represent the minimum, first quartile, median, third quartile, and maximum; the horizontal line reflect the mean of the respective group. Dots represent outliers. *n* = 8 per group

There were no significant changes in MUNIX in either the ADM (Fig. 3a) or TA (Fig. 3b) at HDT4 and HDT59 compared to BDC-5 in any of the groups.

### MUSIX

**Fig. 4:**
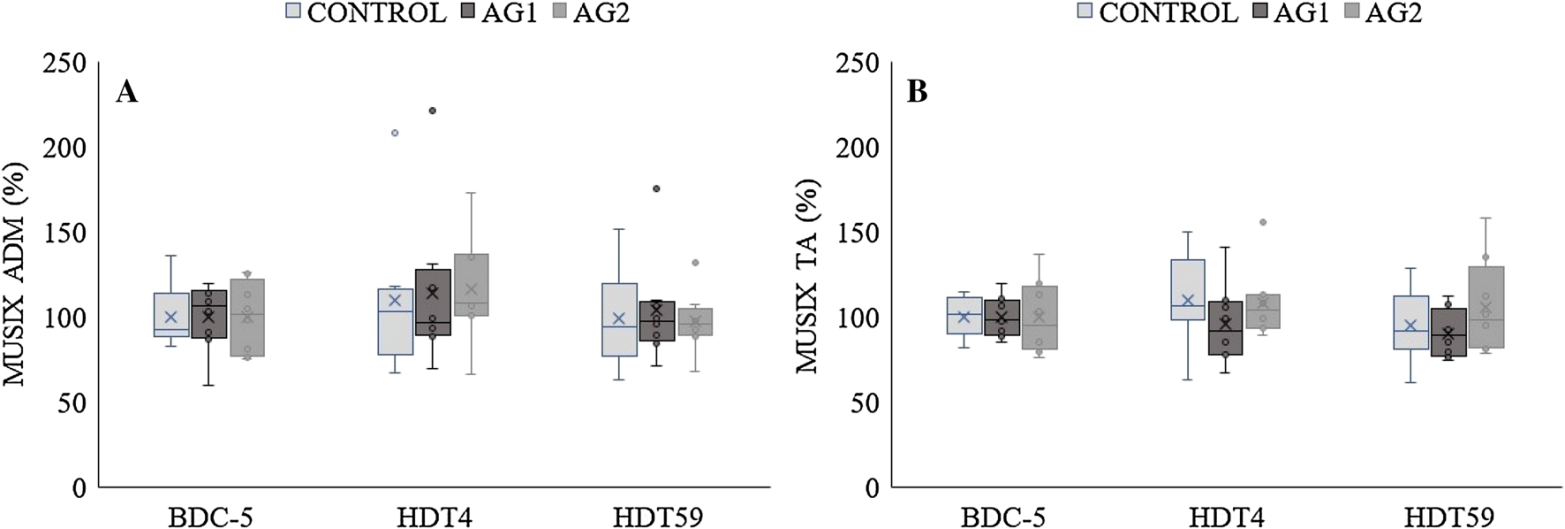
Motor unit number size (MUSIX) of the **a** *m. adductor digiti minimi* (ADM) and **b** *m. tibialis anterior* (TA) before (BDC-5), and at day 4 (HDT4) and 59 (HDT59) of bed rest in CONTROL, AG1 (bed rest + 30 min daily centrifugation) and AG2 (bed rest + 6 × 5 min daily centrifugation). Absolute values from BDC-5 are divided by the group mean to represent 100%. The box plots represent the minimum, first quartile, median, third quartile, and maximum; the horizontal line reflect the mean of the respective group. Dots represent outliers. *n* = 8 per group

In both the ADM (Fig. 4a) and TA (Fig. 4b), there were no significant changes in MUSIX at HDT4 and HDT59 compared to BDC-5 in any of the groups.

## Discussion

The aim of this study was to determine whether MU number and size are altered during long-duration HDBR in healthy individuals and whether AG attenuates any such adaptations. This study was the first to investigate MU number and size during HDBR using the MUNIX method. The main observation of this study is that 60 days of HDBR with or without AG did not cause any significant changes in CMAP, MUNIX, or MUSIX in either the ADM or TA muscles. The absence of significant changes in MUNIX and MUSIX after AG, indicating that AG does not result in MU remodeling, is encouraging and does not preclude application of AG as a countermeasure for disuse-induced physiological deconditioning (Clement 2017).

It was hypothesized that MUNIX decreases during long-duration HDBR based on the previous research observing impairments in MU activation in both upper and lower body muscles during immobilization lasting 4–8 weeks (Seki et al. 2001; Fuglsang-Frederiksen and Scheel 1978). The maintained MUNIX in our study concurs with the absence of significant changes in MFCV; a surrogate measure for MU activation (Mulder et al. 2009), of the *m. vastus lateralis* during HDBR of the same duration as ours. The discrepancy with the other studies may be related to the fact that they used a casting model of disuse. This precludes movement of the joints surrounding the muscle, whereas during spaceflight and bed-based disuse, the joints can move freely (Berg et al. 2007), and merely lack the force of gravity in the longitudinal axis.

Mulder et al. (2009) suggested that the seven testing sessions during their 8-week HDBR were sufficient to maintain neural activation capacity. In our study, however, participants were tested only twice during the HDBR, and it is thus unlikely that the testing sessions prevented changes in MU number or size, particularly when one considers that others have seen that even on the 1st day of leg casting, evidence for reduced MU numbers has been observed (Fuglsang-Frederiksen and Scheel 1978). Moreover, there was no restriction to the degree of possible dorsiflexion, and hand muscles likely worked to a greater extent than in everyday life, due to activities being primarily restricted to upper limb movement, i.e., using a computer, which was probably sufficient to maintain MU activation.

MUNIX has mostly been used to investigate the progressive changes in MU number and size during ALS and ageing, conditions that are characterized by loss of both upper and lower body motoneurons (Sharples and Whelan 2018; Larsson et al. 2019). In general, these studies have observed a 30–42% loss of MUs in the same muscles as tested in our study (Drey et al. 2013; Escorcio-Bezerra et al. 2016), with concurrent increases in MU size (Larsson 2003), due to reinnervation of denervated muscle fibres via nerve sprouting (Gordon et al. 2004; Roy et al. 1996). During space flight, similar losses of MUs have been observed in as short as 14 days in the anti-gravity muscles of space-flown rats (D’Amelio and Daunton 1992), which were recoverable upon return to Earth (Deschenes et al. 2001). Similar findings have been observed in ageing mice, where strength training was shown to prevent the loss of MU numbers, detected by an MUNE method (Giorgetti et al. 2019). Collectively, these results imply, in contrast to what is seen in ageing and ALS, a functional, rather than anatomical loss of MUs. However, equivalent data for humans were hitherto non-existent and our data show that at least during bed rest no such loss of MUs occurs in humans.

It has been observed that the loss of muscle mass and function during HDBR is greater in ankle and knee extensors compared to their flexor counterparts (Alkner and Tesch 2004; Campbell et al. 2019) and even less in upper body muscles (Kamiya et al. 2004). Another study by Dalton et al. (2008) reported similar MU numbers in the soleus of recreationally active old men and activity-matched young counterparts. It is thus possible that the absence of bed rest-induced changes in the MU number and size in the TA and ADM are due to the fact that they are not acting as anti-gravity muscles, and hence are less affected by bed rest than anti-gravity muscles. However, the unaltered MFCV in the *m. vastus lateralis* after HDBR suggests that this is unlikely to be a major explanation for the absence of any bed rest-induced changes in MU number and size of the ADM and TA in our study.

The advantage of MUNIX is that it is easy, time-efficient, and non-invasive. There are, however, some factors that can cause variation between measurements, such as: electrode placement; skin temperature; and ranges of effort during the VICs (Nandedkar et al. 2018). We minimized differences in electrode placement between BDC-5, HDT4, and HDT59 using cellophane sheets with skin and electrode markers for placement of electrodes, and temperature was controlled in the laboratory. In addition, any within-session variation > 10% was excluded from analysis. Although all three assumptions of the MUNIX model, described in “Appendix”, are almost fulfilled for low contractions, MUNIX is still an estimation or approximation of the real number of MUs.

## Conclusion

Using the MUNIX method, we showed that 60 days of HDBR is not associated with significant alterations in MU number and size in the ADM or TA muscles in healthy individuals.

## Appendix: Model for MUNIX (Drey et al. 2013)

Consider an ‘‘ideal’’ muscle with the following three characteristics.

(1) All motor units (MUs) are identical. This means that each Single Motor Unit Potential (SMUP) is identical in amplitude and waveform. (2) The Compound Muscle Action Potential (CMAP) is the sum of all SMUPs. Assuming that there is no temporal dispersion, the CMAP waveform is a magnified image of all SMUPs. (3) Consider a Surface Interference Pattern (SIP) of a voluntary muscle contraction when a few MUs are activated. Assume that their SMUPs do not superimpose.

For the digitalised signal, $u_{i}$ is defined as the amplitude of SMUP at a certain time $t_{i}$. $N$ is the number of motor units contained in the considered muscle. Index $j$ in $u_{ji}$ identifies any SMUP, ranging from 1 to $N$. Therefore, the first assumption can be translated into: $u_{1i} = u_{2i} = u_{i}$. The area of SMUP is computed by adding together the absolute values of $u_{i}$ multiplied by sampling interval $\Delta t$:

$$ {\text{Area}}({\text{SMUP}}) = \sum\limits_{i = 0}^{t} {\left| {u_{i} } \right| \cdot \Delta t} . $$

The power of a signal is computed by squaring its amplitude. Therefore, the power of SMUP is calculated by:

$$ {\text{Power}}({\text{SMUP}}) = \sum\limits_{i = 0}^{t} {u_{i}^{2} \cdot \Delta t} . $$

Assume that $c_{i}$ is the amplitude of CMAP at a certain time $t_{i}$. Taking assumption 2 into account, $c_{i} = N \cdot u_{i}$. To calculate the area of CMAP, the same applies as with the area of SMUP:

1 $$ {\text{Area}}({\text{CMAP}}) = \sum\limits_{i = 0}^{t} {\left| {c_{i} } \right| \cdot \Delta t} = \sum\limits_{i = 0}^{t} {N \cdot \left| {u_{i} } \right| \cdot \Delta t = N \cdot \sum\limits_{i = 0}^{t} {\left| {u_{i} } \right| \cdot \Delta t = N \cdot {\text{Area}}({\text{SMUP}})} } . $$

To calculate the power of CMAP, the same applies as with the power of SMUP:

2 $$ {\text{Power}}({\text{CMAP}}) = \sum\limits_{i = 0}^{t} {c_{i}^{2} \cdot \Delta t} = \sum\limits_{i = 0}^{t} {(N \cdot u_{i} )^{2} \cdot \Delta t} = N^{2} \cdot \sum\limits_{i = 0}^{t} {u_{i}^{2} \cdot \Delta t = N^{2} \cdot {\text{Power}}({\text{SMUP}})} . $$

Consider the area and power of SIP when a few MUs and their corresponding SMUPs $j = D$ are activated (assumption 3):

3 $$ {\text{Area}}({\text{SIP}}) = D \cdot {\text{Area}}({\text{SMUP}}), $$

4 $$ {\text{Power}}({\text{SIP}}) = D \cdot {\text{Power}}({\text{SMUP}}). $$

After some algebraic transformation of (1)–(4), it can be verified that:

5 $$ N = \frac{{{\text{Area}}({\text{SIP}})}}{{{\text{Power}}({\text{SIP}})}} \cdot \frac{{{\text{Power}}({\text{CMAP}})}}{{{\text{Area}}({\text{CMAP}})}} = {\text{ICMUC}}. $$

The aforementioned Eq. (5) reflects $N$ under the condition of the “ideal” muscle. This is indicated by the expression ICMUC (ideal case motor unit count). Nevertheless, the numerical value of ICMUC is quite reasonable given a low force and low SIP area, respectively (see assumption 3). In the model, MUNIX is defined as an ICMUC for an arbitrarily chosen low SIP area (20 mVms), where the assumptions are almost fulfilled. The relation of ICMUC to the SIP area is modelled by a power function:

$$ {\text{ICMUC}} = A \cdot ({\text{Area}}({\text{SIP}}))^{\alpha } . $$

The values of $A$ and $\alpha$ are derived from a regression analysis fitting a power function. Finally, MUNIX is calculated by:

$$ {\text{MUNIX}} = A \cdot (20\,{\text{mVms}})^{\alpha } . $$

## Abbreviations

ADM: Abductor digiti minimi
AG: Artificial gravity
BDC: Baseline data collection
CMAP: Compound muscle action potential
EMG: Electromyography
ESA: European Space Agency
HDBR: Head-down bed rest
HDT: Head-down tilt
MFCV: Muscle fibre conduction velocity
MU: Motor unit
MUNE: Motor unit number estimation
MUNIX: Motor unit number index
MUSIX: Motor unit size
NMJ: Neuromuscular junction
SIP: Surface interference pattern
TA: Tibialis anterior
VIC: Voluntary isometric contraction
